# Defensive Medicine in the Management of Cesarean Delivery: A Survey among Italian Physicians

**DOI:** 10.3390/healthcare9091097

**Published:** 2021-08-25

**Authors:** Vittorio Fineschi, Mauro Arcangeli, Nicola Di Fazio, Zoe Del Fante, Benedetta Fineschi, Paola Santoro, Paola Frati, Associazione Consulcesi Health and ONLUS Futura Ricerca

**Affiliations:** 1 Department of Anatomical, Histological, Forensic and Orthopaedical Sciences, Sapienza University of Rome, Viale Regina Elena 336, 00161 Rome, Italy; nicola.difazio@uniroma1.it (N.D.F.); zoe.delfante@uniroma1.it (Z.D.F.); paola.santoro@uniroma1.it (P.S.); paola.frati@uniroma1.it (P.F.); 2 Istituto di Ricovero e Cura a Carattere Scientifico (IRCCS) Neuromed, Via Atinense 18, 86077 Pozzilli, Italy; 3 Department of Life, Health and Environmental Sciences, University of L’Aquila, 67100 L’Aquila, Italy; mauro.arcangeli@univaq.it; 4 AGI Medica, University of Siena, 53100 Siena, Italy; bfineschi@gmail.com

**Keywords:** defensive medicine, cesarean section, medical responsibility, obstetrics, maternal autodetermination, informed consent, guidelines

## Abstract

Background and Objectives: This study aims to contribute to the definition of the defensive medicine phenomenon between obstetricians and gynecologists, as well as to possible effects on the frequency of deliveries performed by cesarean sections (CS). Materials and Methods: a digital questionnaire was administered through a mail-list including 600 gynecological specialists (of these 168 doctors completed the test), both in public and private settings. It was made of twenty multiple choice questions, concerning their awareness about the practice of defensive medicine and the planning and execution of CS. All doctors involved received clear and complete information about the purpose of this study and about the organizations that received their answers. Analyses of variance and regression were performed to describe differences between groups and to estimate the relationships between variables. The value of *p* < 0.5 was considered statistically relevant. Results: our analysis revealed that most respondents are confident with the defensive medicine definition and characteristics. This survey confirmed that gynecologists fear legal actions promoted by their patients and therefore modulate their choices by implementing professional behaviors of so-called “defensive medicine”. This relates to a greater number of medical liability judgements, which more often concern omission or delayed execution of cesarean section, rather than unskillful surgical procedures. Conclusions: there are few data to support a relation between the high rate of CS and defensive medicine. Numerous scientific studies associated this CS rate with the phenomenon of defensive medicine. This practice is constantly growing in all medical areas, especially in high-risk specialties such as obstetrics and gynecology. Our study highlights physicians’ awareness of adopting defensive medicine behaviors in their clinical practice, affecting the choice of the type of delivery to be performed.

## 1. Introduction

Cesarean section (CS) is the surgical procedure by which one or more fetuses and fetal appendages are extracted through an incision of the abdominal wall and anterior wall of the uterus. The first practices of CS have been recorded between the 4th and 5th century B.C., when this method was considered the only way to save the child when woman was dead or dying [1]. As society has become more and more concerned about patients’ needs, developments in surgical technique have clearly redetermined the way to conceive CS [2]. Considering that CS is a surgical intervention, this method is generally performed in the presence of specific medical indications. However, in the last years, numbers of CS have incredibly increased even in the absence of clinical indications. The international medical community, as reported in the past years by the World Health Organization (WHO), believes that the ideal rate of CS should be between 10% and 15% [3].

The rising of CS rate is a worldwide phenomenon, being the subject of important debates and discussions. In the literature, it has been reported that the rate of CS is increasing both in developed and developing countries [4,5]. Along with other foreign countries, an incredible increase in the number of CS was also recorded in Italy. In particular, data obtained from the 2010 Euro-Peristat Report show that Italy is one of the main countries with the highest rate of CS (around 38%), followed only by Portugal with 33% [6]. The range is between 20 and 25% in countries such as Germany, Ireland, Luxembourg, Hungary, Malta, and Poland, while in Belgium, Czech Republic, Estonia, Latvia, Lithuania, Finland, Sweden, and Norway, it is even less than 20%. The lowest rates are recorded in Slovenia (14.4%) and the Netherlands (15.1%). Substantially, there are marked variations with a lower rate in Slovenia, Nordic countries, Baltic countries, and the Netherlands, while the highest rates are found in southern countries, particularly in Italy.

More recently, the Italian Ministry of Health has published the Annual Report on the birth event in Italy—Certificate of Assistance to the Birth (CeDAP) 2021—which describes the analysis of data collected from the information flow of the CeDAP in 2019 [7].

In this report, the phenomenon of recourse to CS is analyzed through the study of Italian regions based on the Robson classification, recommended by the WHO as a global standard for evaluation, monitoring, and comparing the rate of CS within hospital structures over time and between different hospital facilities [8]. In fact, it has been ascertained that 32.3% of parties took place by CS, with differences between northern and southern regions of Italy. Furthermore, there is a high propensity for the use of CS in accredited Private Hospitals (47.6%), against 30.5% of Public Hospitals.

The main causes are maternal requests, increased maternal age and the phenomenon of defensive medicine. Other factors that influence CS rate, which can vary greatly from country to country, consist of parity, fetus presentation, gestational age, labor mode and any previous CS.

As previously reported, maternal request is one of the causes of increased CS [9,10]. However, the 2011 Guidelines of CS clarify that maternal request, in the absence of clinical reasons, does not specify an indication for CS [11]. The reasons of maternal request for CS are: fear of labor pain, anxiety due to fetal injury/death, fear of childbirth, urinary incontinence, pelvic floor and vaginal trauma, duration of birth, experience of previous labor, anxiety about the loss of control, desire to avoid long labor, anxiety due to lack of support from the staff, fear of defecation, emotional aspects, and anomalies of the prenatal examination [12].

As for the increase in maternal age, the average maternal age at the time of delivery is 32.9 years for Italian women and 30.8 years for foreign citizens. The average age at which Italian women give birth to their first child is over 31, with variations between northern and southern regions. Instead, foreign women give birth to their first child on average at 28.7 years [7].

As far as the phenomenon of defensive medicine is concerned, numerous studies confirm the association between this phenomenon and the execution of CS [13,14,15]. Defensive medicine refers to the practice of recommending a diagnostic test or medical treatment (positive defensive medicine) or avoidance of risky patients or procedures (negative defensive medicine) that serves the function to protect physicians against patients’ claims [16,17]. This practice is constantly growing in the healthcare system, especially in areas characterized by a high risk of complaint, such as surgical areas, gynecology, and obstetrics [17,18,19,20,21].

There is no strong information in Italy on the real incidence of defensive medicine and no certain scientific data about a specific relationship between CS and defensive medicine. In order to implement strategies for improving medical care, it could be useful to investigate the relationship between defensive medicine and CS, as well as the reasons behind it. With the purpose of discovering more about the relationship previously described, in our study we have analyzed the prevalence of phenomena of active and passive defensive medicine in gynecologists and obstetricians. We also studied the reasons behind choice of CS and how this is influenced by maternal choices. Furthermore, we have tried to understand how Italian law will influence the phenomenon of defensive medicine and the use of CS in clinical practice in the next future [22].

## 2. Materials and Methods

A digital questionnaire was created. Subsequently, a mailing list was built by contacting 600 specialists in gynecology and obstetrics registered on the Associazione Consulcesi Health and ONLUS Futura Ricerca portal [23,24]. The specialists selected by the portal possessed the following requisites: (1) both male and female (2) aged between 30 and 70 years; (3) operating on the Italian territory and coming homogeneously from all geographical regions, and (4) operating both in public and private settings.

The questionnaire was developed using twenty questions. To allow higher response rates, a three-point answer scale (Yes, No, Do not know) was provided for each question. This kind of scale was chosen because it is quicker and easier to respond to rather than a five-point or seven-point scale. Questions concerned the following areas:

(1) knowledge of defensive medicine;
(2) attitudes and opinions towards CS;
(3) role of woman’s will and maternal age in performing CS;
(4) role of health facilities and influence of defensive medicine in deciding upon CS;
(5) application of guidelines.

All doctors involved received clear and complete information about the purpose of this study and about the organizations that received their answers (Associazione Consulcesi Health and ONLUS Futura Ricerca).

### Statistical Analysis

Analyses of variance and regression were performed to describe differences between groups and to estimate the relationships between variables. The value of *p* < 0.5 was considered statistically relevant.

## 3. Results

The digital questionnaire was administered to 600 doctors of whom 168 completed the test (the response rate was 28%). Data analysis was conducted based on the gender, age group, and working sector of all responders. Among all responders, 29.21% were aged 30–40 years, 25.43% were aged 40–50 years, 23.87% were aged 50–60 years and 21.49% were aged 60–70. Furthermore, 53.35% of responders were male and 46.35% were female. (Table 1).

Among all the respondents, 112 physicians were working for public hospitals (66.6%), 29 for private hospitals (17.2%), and 27 for both public and private hospitals (16%) (Table 2).

Table 3 shows the questionnaire with relative percentage of answer. Non-respondent ones affirmed that they did not participate voluntarily, due to lack of time or because they thought the questionnaire was useless.

### 3.1. Knowledge and Economic Benefits of Defensive Medicine

As far as defensive medicine is concerned, this study highlights a widespread knowledge on the subject among doctors; in fact almost all the interviewees said they knew what defensive medicine was (85.9%). The second question, “Do you know any specificities of defensive medicine?”, was associated with many positive responses (73.3%). The answers to the following questions, “Do you feel a higher risk of claims?” and “Do you think gynecology is a medical class at higher risk of claims?”, evidence how doctors think that risk of claim in the healthcare system has increased over the years and that gynecology/obstetrics is one of the areas at greatest risk (99.41 and 98.81). Furthermore, doctors practice defensive medicine as evidenced by the majority of positive answers on these topics. The results of the questionnaire show a greater application of negative defensive medicine (87.51%) than a positive one (72.7%), although both are frequently performed.

### 3.2. Attitudes and Opinions towards CS

This part of the questionnaire investigates gynecologists/obstetricians’ preferences with respect to CS and natural delivery. About 91% of respondent prefer to perform CS compared to vaginal delivery. Furthermore, 80% of the interviewed doctors believe that spontaneous delivery is more risky than CS. Otherwise, only 19% of respondent think that vaginal delivery is safer than CS. Certainly, the analysis of these answers must consider what has been happening for several years in terms of criminal and civil trails against obstetricians in the management of childbirth. About the increase in the rate of CS and their connection to the phenomenon of defensive medicine, the analysis of case law has shown the presence of a greater number of judgments condemning the doctor for failure to perform, or delay in performing, deliveries by cesarean section, rather than cases of liability for surgical errors during the delivery. This attitude of the judges was considered by the doctors, with perhaps superficial reflection, as a reason to consider CS safer and with less litigation as a result.

### 3.3. Role of the Mother’s Will and the Maternal Age in Performing CS

The third area of the questionnaire examines how the mother’s will and maternal age influence types of birth. In fact, 79.77% of respondents thought that mothers would influence types of birth. This trend led to an increase in CS even in the absence of clinical indications. In fact, the guidelines do not give any indication about following maternal will. Besides, 76.79% of respondents think that higher maternal age is a factor that increases resort to CS.

### 3.4. Role of Health Facilities and Influence of Defensive Medicine in Deciding upon CS

This section of the questionnaire highlights how defensive medicine affects the choice of type of birth. In fact, 92.37% of respondents confirmed that one of the main causes of the higher rate of CS is attributable to defensive medicine. Furthermore, 92.36% of those interviewed said that CS is performed more in private hospitals than in public hospitals.

### 3.5. Application of the Guidelines

Both the Italian and international guidelines’ knowledge in 97% of respondents was up to date [25,26]. The current Italian guidelines state: “admission to labor, in the absence of specific contraindications, should be offered to all women who have already given birth by CS. In relation to the increased absolute risk of uterine rupture, the possibility of vaginal delivery after CS is contraindicated in cases of previous rupture of the uterus, previous longitudinal uterine incision, and three or more previous CS.

Women who have already given birth by CS should be provided with adequate clinical surveillance and continuous electronic fetal monitoring in the active phase of labor. The healthcare facility should ensure immediate access to the operating room and resuscitation and ready availability of blood transfusions in the event of an emergency cesarean section.

Health professionals should provide the woman with a previous CS with information about the likelihood of vaginal delivery based on her medical history and the hospital’s case history. In addition to clinical information on the mode of delivery, women with a previous CS should be provided with information on the characteristics and organization of the facility and specific information on the mode of care in use (induction of labor, use of oxytocin, use of analgesia, use of prophylactic vaginal operative delivery), as these aspects may affect maternal and fetal/neonatal outcomes” [25].

About 74% of respondents think that guidelines should address gynecologist/obstetricians when choosing mode of delivery. However, about 20% of respondents affirm that guidelines might not always indicate the correct type of delivery to be performed. About 63% of interviewed admitted that, due to maternal requests, they did not follow guidelines’ recommendations, while others (about 70%) do not apply guidelines to avoid health claims.

Finally, in the last question, 63% of respondents affirm that actual Law No. 24/2017 (the so-called Gelli-Bianco Law) about medical responsibility will reduce claims in gynecology and obstetrics [22]. The law states that the doctor can only be held criminally liable in cases of gross negligence and that in civil law it is mainly the hospital that must pay compensation for any damage to the mother and child [27]. The law tackles the mounting concern over litigation related to medical malpractice and calls for Italian physicians to follow guidelines. The law provided for the decriminalization of simple negligence of a physician on condition that he/she followed the guidelines and “good medical practice” while carrying out his/her duties, whilst the obligation for compensation, as defined by the Italian Civil Code, remained [27].

## 4. Discussion

The present research aims to evaluate and analyze Italian physicians’ knowledge, including their direct experience, about the practice of defensive medicine and how it influences CS rate. The incidence of CS appears to be globally increasing, as of the Euro-Peristat report, as well as the certificate of childbirth assistance (CEDAP) data. WHO claims that since 1985 about 15% of cesarean sections were performed without any medical advice. An increase in maternal age, mothers’ will, and defensive medicine are the main cause determining the increased ratio of cesarean sections.

Clearly, as Italian law provides for any medical procedure, CS can be carried out only after receiving informed consent from the patient [28].

Defensive medicine occurs when tests, medical examinations and high-risk procedures are performed in order to avoid the risk of malpractice medical claims [29,30,31]. Defensive medicine represents a global phenomenon which highly impacts costs in healthcare [32,33,34]. Indeed, performing instrumental or laboratory tests without medical indication leads to longer waiting lists and an increase in healthcare system spending [35,36,37,38].

The results of our study highlighted a prominent focus of obstetrical specialists about defensive medicine. In fact, our analysis revealed that most of the respondents were confident with the defensive medicine definition and characteristics. Many interviewed physicians affirmed they personally experienced defensive medicine, 72.7% of them reported to carry out positive defensive medicine, while 87.51% admitted practicing negative defensive medicine. When we asked participants whether the practice of defensive medicine might affect insurance costs, a large part of them answered positively (69.6%). These findings led us to suppose that there is general awareness, among physicians, about the cost of defensive medicine. Thus, it can be assumed that most physicians consider defensive medicine as the best way to protect themselves from medical claims.

In order to quantify numerically the predisposition of gynecologists to defensive medicine, a further analysis of the data was conducted.

**Figure 1:**
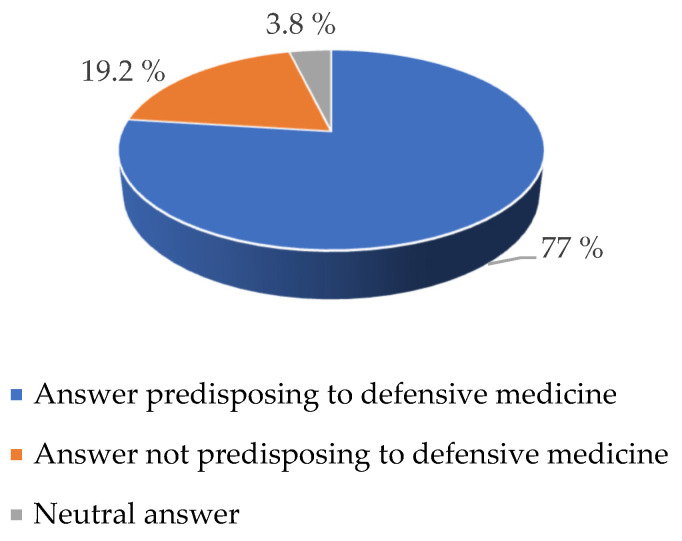
Percentage of answer predisposing to defensive medicine.

The answers to the questions of the survey that were closely focused on the theme of defensive medicine were examined (question number 5, 6, 7, 18, and 19). The results of this analysis are expressed in Figure 1.

It emerged that 77% of the responses provided were predisposed to defensive medicine, while only 19.2% were not predisposed to defensive medicine. A small percentage (3.8%) of responses took a neutral attitude towards defensive medicine itself. Based on these data, it can be said that concerning the problems of CS, a substantial percentage of doctors interviewed (77%) were well aware of the problems related to defensive medicine and oriented their attitude in relation to this.

The high admission rate of defensive medicine behaviors has been confirmed in several surveys [13,39]. In a study conducted in three English hospitals (two in South Wales and one in Kent), a high percentage (78%) of defensive medicine has been reported, with a major rate for positive form (59%) rather than negative ones (9%) [40]. Compared to these findings, our data showed a major propensity for avoidance behaviors (87.51%). Almost all the respondents declared that today, doctors are at higher risk of complaints from patients (99.41%).

As regarding the application of defensive medicine in the gynecological field, it is necessary to consider this area as one of the most frequently involved in medical liability. In our analysis, we found that the majority of physicians (98.81%) believe that obstetrics and gynecology are specialties particularly at risk for complaints.

In obstetrics, defensive medicine expresses through an increasing rate of CS rather than natural delivery because CS, despite its surgical nature, is thought to be a safer procedure [41,42].

In our analysis, we investigated physicians’ perception about the riskiness of CS in comparison to spontaneous delivery. We found that 19% declared that cesarean delivery is riskier than spontaneous delivery and 81% denied this statement. These data demonstrate that there is no direct perception about the mortality and morbidity of CS and how this method is becoming a consolidate practice.

As the number of CS on request has been increasing among Western countries, the opportunity to allow requests for CS without medical recommendations has become both a clinical and ethically important dilemma [43]. An interesting meta-analysis has been written by Mozurkewich EL and Hutton EK, enlightening that induced labor may result in small increases in uterine rupture rate and in fetal and neonatal mortality rates rather than elective repeat cesarean delivery [44].

As a result of this orientation, Italy has seen a crisis in the insurance market. Insurance premiums have increased, because judges in medical malpractice cases concerning newborns tend to decide up to large compensations (up to EUR 5 million) which are usually higher than insurance coverage. Insurance premiums can reach up to EUR 10 thousand per year depending on the risk of different specialties, such as obstetrics and gynecologist, while general physicians pay a much lower premium.

In the United States, Japan, New Zealand, and other European countries, to reduce medical liability, a no-fault compensation system has been adopted [45,46]. In some circumstances, compensation can be paid without proven negligence, thus clearly leading to an attempt in deflating litigation and containing defensive medicine, aiming to protect both patients and physicians [47].

Finally, it is of paramount importance to note that repeat CS accounts for a large percentage of total CS. Meanwhile, it is remarkable that vaginal birth after cesarean (VBAC) could represent an important option for reducing the CS rate. In the obstetrical practice in the 20th century, the recourse to CS was an obligatory choice following a CS due to the belief that “once a cesarean section, always a cesarean section” [48]. Nowadays, even though vaginal birth has emerged to be safe following CS, repeat CS still permeates the routine of a large number of professionals and services [49]. To promote VBAC, thus reducing the CS rate, factors that influence the decision-making process have been investigated. Recent studies showed that informed choice, which includes informed recovery from a surgical birth, has primary importance in relation to decision-making for women [49]. Thus, a model of care that includes prenatal counseling and balanced information about VBAC is needed [50,51].

## 5. Conclusions

In conclusion, our study confirmed that doctors are aware of adopting defensive medicine behaviors in their clinical practice and that this affects the choice of type of delivery to be performed. Thus, in this context, clinical guidelines are usually not considered in the decision process.

Our study has limitations such as the low number of specialists who responded to the questionnaire. In addition, the questionnaire is single-answer, and we did not use a Likert scale for fear of even lower adherence by physicians.

However, new legal provisions are expected to modify this phenomenon in the next year, in Italy. In fact, the rate of CS is likely to be reduced due to the limitations imposed by law, provided that healthcare professionals should always be compliant to guidelines approved by National Health Care System. Furthermore, these recommendations protect healthcare professionals in case of medical–legal disputes.

Further investigation needs to be conducted to assess the role of the health care professional in the VBAC decision-making process.

## Figures and Tables

**Table 1:** Participants’ general information.

Participants	Age (Years)				Sex (%)	
	30–40 years	40–50 years	50–60 Years	60–70 years	Male	Female
Respondent	29.21%	25.43%	23.87%	21.49%	53.35%	46.35%
Non-respondent	20.37%	24.45%	23.32%	31.86%	52.11%	47.89%

**Table 2:** Participants’ working sector information.

Participants	Type of Working Sector (%)		
	Public Hospitals	Private Hospitals	Both Public and Private hospitals
Respondent	66.6%	17.2%	16%

**Table 3:** The questionnaire.

	Yes	No	Do Not Know
(1) Do you know what defensive medicine is?	85.9%	14.1%	0%
(2) Do you know any specificities of defensive medicine?	73.3%	21%	5.7%
(3) Do you think there is a higher risk of claims?	99.41%	0.59%	0%
(4) Do you think gynecology is a medical class at higher risk of claims?	98.81%	1.19%	0%
(5) Have you ever performed any positive type of defensive medicine?	72.7%	19.6%	7.7%
(6) Have you ever performed any negative type of defensive medicine?	87.51%	10.35%	2.14%
(7) Do you think that gynecologists/ostetricians prefer Cesarean Sections (CS) rather than the natural ones?	91.4%	5.6%	3%
(8) Do you think that natural childbirth is more dangerous than the CS?	80%	20%	0%
(9) Do you think that the CS is more dangerous than the natural childbirth?	19%	81%	0%
(10) Do you think that women’s will can influence their choice to resort to a CS?	79.77%	16.07%	4.16%
(11) Do you think that defensive medicine affects the cost of insurance?	69.6%	26.3%	4.1%
(12) Do you think that there can be a link between the percentage of CS and the internal organization of the places where births take place?	86.91%	9.52%	3.57%
(13) Do you think that the percentage of CS increases when the age of the mothers is higher?	76.79%	22.02%	1.19%
(14) Do you think that the percentage of CS is higher in the private sector?	92.36%	4.07%	3.57%
(15) Do you think that the high percentage of CS in Italy is strictly related to defensive medical phenomena?	92.37%	4.67%	2.96%
(16) Do you know the last guidelines about CS?	97%	3%	0%
(17) Do you think that the guidelines can help the specialists to make appropriate choices regarding the CS?	74.42%	17.85%	7.73%
(18) Have you ever been far from the instructions given by the guidelines as far as the mothers’ requests are concerned?	63%	37%	0%
(19) Have you ever decided not to follow the instructions given by the guidelines because of the fear of health complaints?	71%	24%	5%
(20) Do you think that the Italian law can reduce the health litigations?	63%	25%	12%

## Data Availability

The data that support the findings of this study are available on request from the corresponding author. The data are not publicly available due to privacy or ethical restrictions.

## Abbreviations

CS: Cesarean section; WHO, Word Health Organization; CeDAP, Certificate of attendance at childbirth.
